# Assessment of Cementitious Ceramic Tile Adhesives in the Light of Repeatability and Reproducibility of the Tensile Adhesion Strength Measurements

**DOI:** 10.3390/ma16124245

**Published:** 2023-06-08

**Authors:** Jacek Michalak, Radosław Ziomek

**Affiliations:** Research and Development Center, Atlas sp. z o.o., 2 Kilińskiego St., 91-421 Lodz, Poland

**Keywords:** tensile adhesion strength, ceramic tile adhesive (CTA), assessment and verification of constancy of performance (AVCP), construction products, repeatability, reproducibility

## Abstract

The paper presents the results of tensile adhesion strength measurements of ceramic tile adhesive (CTA) stored in various conditions performed by ten operators in one laboratory using the same equipment and auxiliary materials. The obtained results allowed the authors to estimate the repeatability and reproducibility of the tensile adhesion strength measurement method using the methodology following ISO 5725-2:1994+AC1:2002. Standard deviations of repeatability ranging from 0.09 to 0.15 for the general means value in the range of 0.89–1.76 MPa and standard deviations of reproducibility ranging from 0.14 to 0.21 for the same general means content indicate that the accuracy of tensile adhesion strength measurement method is not high enough. From the group of ten operators, five perform tensile adhesion strength measurements daily, the remaining five perform other measures, and the results obtained by professionals and non-professionals showed no significant differences. In light of the obtained results, compliance assessment with this method with the criteria set out in the harmonized standard EN 12004:2007+A1:2012 carried out by different operators may be divergent, and there is a significant risk of incorrect assessments. This risk is additionally increasing in the case of the evaluation conducted by market surveillance authorities, which use the simple acceptance rule that does not consider measurement variability.

## 1. Introduction

Ceramic tiles adhesives (CTAs) used to attach ceramic tiles inside and outside buildings are changing. Currently, the use of thin-bed CTAs involves complex multi-component systems, the significant development of which is related to the dry-mortars technology achievement [1,2]. In 2001, the European established standard EN 12004, which unified the requirements for CTAs and introduced the two classes, C1 and C2 [3]. Setting the EN 12004 standard was crucial in developing the CTA market. Previously, different European countries had other requirements, and in addition, many manufacturers declared the properties of their products in different ways. Another critical stage in the CTAs market development was the International Organization for Standardization (ISO), establishing the ISO 13007 series of standards with the exact requirements previously set by CEN [4]. Thus, the requirements for CTAs have gained a global dimension. It is essential to consider the market size for these products, which is estimated to be around 65 million tons annually [5].

In the case of construction products, it is essential to define their characteristics so that their fulfillment secures the daily stress of their use. For cementitious CTAs, bond strength and durability of bond strength against specified external factors are particularly important. For this reason, adhesion strength measured by tensile testing is the basis for evaluating cementitious ceramic tile adhesives [6]. By subjecting the CTA to the assessment and verification of constancy of performance (AVCP), the manufacturer determines the bond strength and the durability of the bond strength in the system: concrete substrate (slab)—CTA—ceramic tile as initial adhesion, adhesion after thermal aging, adhesion after immersion in water, and adhesion after freeze–thaw cycles, as well as in terms of reaction to fire and release of hazardous substances [6].

From the perspective of over two decades of assessing CTAs following the requirements of EN 12004 or ISO 13007, it is essential to point out that the unification of requirements had a very positive impact on the market [2]. Despite the advantages noted with the advent of EN 12004, articles and reports pointed to problems in the detailed assessment of CTAs related to the imperfections of tensile adhesion strength measurement. Thus, Felixberger, in his research, mentioned that from the physical standpoint, the shear stress measurement would be more appropriate for the CTAs’ assessment, primarily due to the actual operating conditions of these products [7]. In the same research, he indicated the effect of concrete slab type on the results of tensile adhesion strength measurements [7]. The following years provide reports on the influence of ceramic tiles, their underneath relief [8], and the type of water used to condition the samples [9] on the tensile adhesion strength value. In both cases, the observed differences were so significant that they determined whether or not CTA met the declared properties. Other tests have shown the effect of auxiliary materials, the location of the tested joint on the concrete slab [10], and the procedure during the sample preparation for measurement [11] on the results of measuring adhesion.

Due to the variability associated with the measurement of adhesion using the pull-off technique for construction products other than CTAs [12,13,14], the differences between the tensile adhesion strength measurements of CTAs under laboratory and real-life conditions [15,16,17,18], as well as other influencing factors on the course and the final measurement result [19], doubts are raised as to the possibility of a reliable CTA assessment under certain conditions only through the tensile adhesion strength measurement [5,20,21]. There are postulates that the current model of CTAs assessment using the tensile adhesion strength measurement should be modified by adding an evaluation in the AVCP process concerning another characteristic [11,20,21].

Completely independent of the studies mentioned above, interlaboratory comparisons (ILCs) of tensile adhesion strength of CTAs were performed by Cerprocim SA [5,22,23,24]. ILCs have been conducted and are, above all, to assess the competence of laboratories [25,26] concerning the accreditation requirements of accreditation bodies following EN ISO/IEC 17025:2017 [27]. The z-score analysis under the criteria specified in EN ISO/IEC 17043:2010 describes and assesses the professionalism of the laboratories participating in the ILCs [24]. Although the standard deviation of reproducibility of the method reached even 40% in some cases, the results obtained from almost all laboratories are considered satisfactory [5]. The analysis of the results also confirmed that constant participation in ILCs improves the quality of laboratory operations [22,25,26]. The first ILC of CTAs’ open-time measurement took place in 2021. The participants measured open time after 30 min by measuring the tensile adhesion strength. This characteristic of CTA—defined as the maximum time after applying the CTA when ceramic tiles are embedded in the adhesive layer to obtain the required adhesion—is a crucial feature, essential from the perspective of the tiling contractor. The correct open time allows the contractor to correct the tile’s position without reducing adhesion. When measuring the open time, the ILC showed that the reproducibility standard deviation is as high as 75% [28]. 

Another third dimension regarding assessing CTAs from the perspective of measuring tensile adhesion strength is inspections by market surveillance authorities. Inspections are in EU countries, a part of permanent market surveillance regulated by EU law [29]. Market surveillance authorities conduct inspections to verify the correctness of the CE marking (CTAs are placed on the market under conformity assessment system no. 3) and subject construction products to laboratory tests to confirm the product characteristics declared by the manufacturer. For CTAs, this performance property is the durability of the bond determined by measuring tensile adhesion strength following the test method specified in EN 1348:2007 [30]. 

In Poland, when reassessing CTAs, market surveillance authorities apply the simple acceptance rule that does not consider the variability resulting from measurement uncertainty [31,32]. This approach to the issue is the cause of significant discrepancies in the assessment of CTAs by their producers compared to market surveillance authorities [21]. In addition, since the standard deviation of reproducibility for tensile adhesion strength measurements is high, divergent results obtained in different laboratories cause additional tensions between manufacturers and market surveillance authorities [5,33].

It would seem that the issues of repeatability and reproducibility of measurements are well known [34,35]. However, few studies focus on the repeatability or reproducibility of measurement methods used to assess construction products [33,36]. Furthermore, problems with repeatability and reproducibility are ubiquitous in various fields of human activity. In the case of many construction materials, the complexity of the processes related to the multi-stage nature of the testing procedures is not sufficiently considered. The complexity level and interconnections between the individual stages of research do not facilitate improvement in terms of repeatability and reproducibility of measurement methods [33,36,37]. In addition, an important aspect is the awareness of the purposes for which the measurement method is or will be used [38].

As a rule, standards are guidelines or criteria used to ensure consistency, quality, and product comparability by defining the basis for testing. An important aspect is that the test methods used in the standards are validated beforehand to ensure that they are suitable for the intended uses. The main objective of all these endeavors is to provide an appropriate level of assurance to all stakeholders, mainly customers.

This paper analyzes the results of tensile adhesion strength measurements obtained for the same CTA by ten operators working in the same laboratory using the same equipment and auxiliary materials. The work aims to show the complexity of measuring tensile adhesion strength and the risk associated with assessing CTAs based on counting only this parameter. Results obtained in this study made it possible to determine the repeatability and reproducibility (intra-laboratory) of the CTAs’ tensile adhesion strength measurement method. The tests and the results obtained are presented to show the complexity of standardization, in this case, to show the adequacy of using the adhesion measurement method for product evaluation. The adequacy of the method used is an essential aspect of standardization, which is a challenge. 

## 2. Materials and Methods

The study used a CTA classified by its manufacturer as class C2 following EN 12004:2007+A1:2012. The operators participating in the study marked the CTA residue on the sieve with a mesh size of 310 μm. The average sieve residue and the reproducibility standard deviation were calculated based on the results. All obtained results were in the range ±1 s, where s is the reproducibility standard deviation.

In the tensile adhesion strength of the CTA procedure, an adhesive layer is applied to a concrete slab. Then, after a specific time, ceramic tile is laid on the CTA layer, which is loaded with a particular force for an exact time—all these activities are performed per the procedure described in clause 8.1. of EN 1348:2007. The prepared test sample, consisting of a concrete slab—CTA—ceramic tile, is seasoned. Then, the sample tensile holder is glued to the ceramic tile, and after further storage of the sample, the adhesion of the CTA is determined by applying a force increasing at a constant speed. All these activities are performed following the procedures described in clauses 8.2.–8.5. EN 1348:2007. Results were calculated according to the requirements of clause 9. EN 1348:2007.

The tests used the ceramic tile and concrete slab that met the requirements specified for these materials in EN 1348:2007. It is worth noting that the concrete slab used in the tests was considered the most appropriate in the environment of CTAs producers, and the Solana concrete slab was manufactured by Mosaicos Solana S.A., Spain. The concrete slab complied with the requirements of EN 1323:2007 [39].

Tensile adhesion strength CTA tests were performed by ten operators, five of whom perform these tests daily and five operators who perform tests other than tensile adhesion strength measurements in their daily work. Table 1 presents the data characterizing the operators who performed the tests described in this paper.

The selection of operators was made using one of the non-probability selection techniques—purposeful selection (arbitrary, discretionary, selective), which is the most typical case of non-random selection [40]. In the authors’ opinion, despite the operators’ subjective choice, such a choice provided valuable information.

## 3. Results

Table 2, Table 3, Table 4 and Table 5 present the measurement results of initial tensile adhesion strength, tensile adhesion strength after immersion in water, tensile adhesion strength after heat aging, and tensile adhesion strength after freeze–thaw cycles obtained by ten operators performing the tests. In addition, the table specifies the calculated mean value from a series of measurements performed by each operator. The measurement series mean value was calculated, following the recommendations of clause 9 of EN 1348:2007. At first, the operator enumerated the mean value from ten measurements and then discarded the values that differ by more than ±20% from the mean value. For the remaining values (must be a minimum of five values remaining), a new mean has been determined. The tables show the standard deviation calculated from a series of results after rejecting those values that differ by more than 20% from the mean value calculated from all ten measurement results. 

All calculated data were recorded to one more significant figure than the test results values following the recommendation of ISO 5725-2:1994+A1:2002 [41].

Essential in the analysis is the reference of the standard deviation to the mean value of the measured feature. The tables also show the values of the standard deviation expressed as a percentage, a measure of variability that allows estimating the share of the error resulting from the dispersion in the determined mean value of the tested feature.

Two groups of operators carried out the study. The first group consisted of non-professionals (operators marked with the symbols A, B, C, D, and E) employed in the laboratory where the measurements described in this paper were carried out but performed tests other than tensile adhesion strength daily. The second group consisted of five people (operators marked with the symbols F, G, H, I, and J) regularly performing tensile adhesion strength tests. Table 2, Table 3, Table 4 and Table 5 also present the standard deviation of measured value expressed as a percentage characterizing the group of professionals and non-professionals and the set of all operators.

In the case of tensile adhesion strength measurements, it is also essential to provide the model of failure recorded due to the measure. Table 2, Table 3, Table 4 and Table 5 show the failure model following the systematics given in EN 12004:2007+A1:2012.

## 4. Discussion

**Figure 1:**
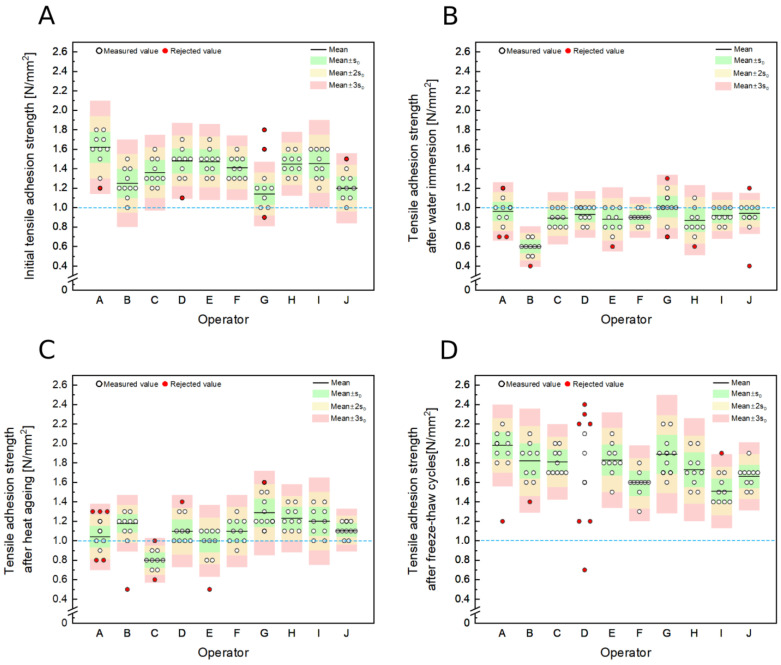
The initial tensile adhesion strength (**A**), tensile adhesion strength after immersion in water (**B**), tensile adhesion strength after heat aging (**C**), and tensile adhesion strength after freeze–thaw cycles (**D**) of CTA measurement results obtained by ten operators participating in the study. The threshold value of 1 N/mm^2^ (CTA class C2 according to EN 12004:2007+A1:2012) is marked with a blue dashed line (---).

Figure 1 shows the measurement results of initial tensile adhesion strength, tensile adhesion strength after immersion in water, tensile adhesion strength after heat aging, and tensile adhesion strength after freeze–thaw cycles obtained by all ten operators participating in the study. The mean value and the ranges of values corresponding to mean value ± *s_D_*, mean value ± 2*s_D_*, and mean value ± 3*s_D_*, have been marked in the figures for each series of measurements performed by each operator. As we know, in the case of a normal distribution, 68.2% of the results are in the mean value ± *s_D_* range, 95.4% are in the mean value ± 2*s_D_* range, and 99.8% of observations are in the mean value ± 3*s_D_* range. The figures also indicate the results rejected following the requirements specified in clause 9 of EN 1348:2007.

In the initial tensile adhesion strength case, the lowest mean value was 1.23 N/mm^2^ (operator J), and the highest was 1.58 N/mm^2^ (operator A). In the case of the determination of tensile adhesion strength after immersion in water, the results ranged from 0.58 N/mm^2^ (operator B) to 1.01 N/mm^2^ (operator G). Operator C obtained the lowest mean value of 0.80 N/mm^2^, and operator G got the highest mean value of 1.32 N/mm^2^ for tensile adhesion strength after heat aging. The last measurement—tensile adhesion strength after freeze–thaw cycles—was characterized by values ranging from 1.55 N/mm^2^ (operator I) to 1.98 N/mm^2^ (operator A). In the case of tensile adhesion strength after freeze–thaw cycles measurement, one of the operators—operator D, obtained results, of which as many as seven did not meet the condition specified in clause 9 of EN 1348:2007. For this reason, there was no further analysis. 

Comparison of the *s_D_*/*m* ratio in two groups—non-professionals and professionals showed that professionals obtained more consistent results, characterized by a smaller scatter than the results obtained by non-professionals. In the initial tensile adhesion strength case, the value of *s_D_*/*m* was 9.1% for F–J operators and 9.8% for non-professionals. In the case of the determination of tensile adhesion strength after freeze–thaw cycles, the non-professionals group obtained a lower *s_D_*/*m* value than the professionals. However, one should remember that non-professional—operator D—got results characterized by such a wide scatter that it was impossible to include it in further analysis, taking into account the requirements of clause 9 of EN 1348:2007.

The analysis of failure patterns clearly showed a high consistency of the obtained results. In the case of initial tensile adhesion strength, adhesive failure between tile and adhesive (AF-T) occurred 99 times, and cohesive failure within the adhesive (CF-A) only once. In the case of the determination of tensile adhesion strength after immersion in water, adhesive failure between the tile and pull head plate (BT) was recorded six times, and AF-T was in the remaining cases. After heat aging, 91 AF-T was obtained for tensile adhesion strength, eight times CF-A, and once a hybrid system—AF-T/CF-A. In the case of the last measurement—for tensile adhesion strength after freeze–thaw cycles, the CF-A failure pattern occurred 92 times, BT three times, AF-T twice, and a mixed CF-A/AFT failure three times.

The CTAs adhesion measurement method repeatability and reproducibility were determined, following the recommendations of ISO 5725-2:1994+AC1:2002 [41]. The statistical model adopted in the above standard is suitable for test results analysis and interpretation, the distribution of which is approximately normal. During the execution of the experiment, all basic rules given in the standard are followed; each laboratory/operator receives the same number of test results, and each laboratory/operator takes into account the same levels of the test sample. It is the so-called balanced experiment with equal levels.

In the case of the CTAs adhesion measurement described in the article, the obtained results have an approximately normal distribution. The use of the Shapiro–Wilk test for testing the similarity of the distribution of a given variable to the normal distribution, in the case of using test results given with an accuracy of one decimal place (results presented in Table 2, Table 3, Table 4 and Table 5), showed that only in the case of initial tensile adhesion strength the results obtained by all operators have a normal distribution. In the case of measuring tensile adhesion strength after immersion in water, for the results obtained by five operators (C, D, F, I, and J), normality can be rejected. In the case of measuring tensile adhesion strength after heat aging, the results of four operators (B, D, E, and J), and in the case of measuring tensile adhesion strength after freeze–thaw cycles, the results of three operators (A, C, and F) also vary from the normal distribution. In a situation where instead of the results given with an accuracy of one decimal place in the Shapiro–Wilk test, results with an accuracy of two decimal places were used, the number of results that do not meet the normality will decrease, which is to be expected. However, the results obtained by operator C for tensile strength after immersion in water, the results of operators D and F in the case of tensile adhesion strength after heat aging, and the results of operator A for tensile adhesion strength after freeze–thaw cycles are not within normal distribution. Thus, the condition required to be used for the analysis and interpretation of the results of parametric tests, such as ANOVA, was not met.

In addition, the methodology described in ISO 5725-2:1994+AC1:2002 is justified due to its widespread use to determine the repeatability and reproducibility of measurement methods. 

For each of the four measurement methods, i.e., initial tensile adhesion strength, tensile adhesion strength after immersion in water, tensile adhesion strength after heat aging, and tensile adhesion strength after freeze–thaw cycles), the precision of the method was determined using repeatability standard deviation (*s_r_*), reproducibility standard deviation (*s_R_*) with the indication of the general mean ($\hat{m}$). Due to minor differences between the results obtained in the group of professionals and non-professionals, in calculating the method’s precision following ISO 5725-2:1994+AC1:2002, all results (professionals and non-professionals) were used. The calculation results are presented in Table 6.

One cannot always take for granted that there is a regular functional relationship between the precision of the test method and the general mean ($\hat{m}$). In the examined case, both for the standard deviation of repeatability and the standard deviation of reproducibility, based on the results presented in Table 6, it is possible to describe these functional relationships with a straight line with a positive intercept. However, since only four data pairs for repeatability and four data pairs for reproducibility were obtained as a result of the tests, and taking into account that tensile adhesion strength was determined each time, but after different storage conditions of the sample, and the complexity of the measurement procedure and the heterogeneity of the tested material affects measurement variability, another functional relationship may describe this test method.

In all studied cases (initial tensile adhesion strength, tensile adhesion strength after immersion in water, tensile adhesion strength after heat aging, and tensile adhesion strength after freeze–thaw cycles), we obtained *s_r_*/*s_R_* < 1, which means underestimated uncertainty in simplified consideration.

Previously conducted ILCs showed that the standard deviation of reproducibility for tensile strength, tensile strength after immersion in water, tensile strength after heat aging, and tensile strength after freeze–thaw cycles of CTA ranges from 0.22 to 0.60 [5,33]. The results obtained in this study, presented in Table 6, showed that in the conditions of the same laboratory, the use of the same auxiliary materials (concrete slab, ceramic tile, measuring devices, water for sample preparation and conditioning), the standard deviation of reproducibility is half lower than in case, interlaboratory using different concrete slabs, different ceramic tiles, different measuring devices, water although concrete slabs, ceramic tiles, and pull-off tester meet the requirements specified in the EN 1348:2007 standard. 

The values of the standard deviation of reproducibility and repeatability determined in this paper, amounting to 0.09–0.15 and 0.14–0.21, respectively, are higher and significantly higher than the standard deviation of reproducibility and reproducibility for other methods used to assess construction products, such as compressive strength measure or thermal transmittance [33].

The risk of making an incorrect decision related to the uncertainty of test results in the case of construction products is not often the subject of scientific articles [33]. A too-high uncertainty value means the method is inadequate for product assessment. Scientific papers on standardization are written primarily by representatives of the academia, to a lesser extent by practitioners [42,43]. There is a widespread belief that scientists are experts. For this reason, it is crucial that publishing results showing the inadequacy of solutions, as in the case of the results described in this article, fills in a missing link of limited knowledge transfer from industry to academia. 

## 5. Conclusions

In the studies described in this paper, the authors determined the repeatability and reproducibility standard deviation of tensile adhesion strength measurements of CTAs after different sample storage conditions. The standard deviation of repeatability and reproducibility results show that the tensile adhesion strength measurement method characterizes significant variability. The values of the standard deviation of repeatability ranging from 0.09 to 0.15 and the standard deviation of reproducibility (in intra-laboratory conditions) in the range from 0.14 to 0.21 indicate significant limitations with the use of this method. In particular, assessing and verifying the constancy of the performance of CTAs using a simple acceptance rule does not consider the variability resulting from measurement uncertainty.

Comparing the results obtained by the group of professionals versus non-professionals participating in the study showed that the results obtained by professionals were characterized by less variability, although the differences were not significant.

The values of the standard deviation of repeatability and standard deviation of reproducibility determined in this study reinforce the views that the measurement of tensile adhesion strength should not be the only one when assessing CTAs. In addition, the obtained results confirm that the measurements of some construction products, due to the complexity of sample preparation and their heterogeneity, are subject to too high variability, and their assessment is complex.

In addition, the designated standard deviations of reproducibility and repeatability signal a possible revision of the tensile adhesion strength measurement method. It is necessary, in each case when a standard is created, to validate the test method. Due to the safety of the products, which is vital for all stakeholders, it would be reasonable to determine at what level of reproducibility and repeatability the method can be used to evaluate the product while indicating in which case it is possible to use a simple acceptance rule by market surveillance authorities.

## Figures and Tables

**Table 1:** Characteristics of operators who participated in the tensile adhesion strength of CTA tests.

Operator	Sex	Age	Performing Tensile Adhesion Strength Measurements Daily	Work Experience in the Laboratory Participating in the Study [Years]	Total Work Experience in Laboratory [Years]	Education
A	M	50	no	26	26	M.Sc.
B	F	35	no	11	11	M.Sc.
C	M	36	no	7	12	Ph.D.
D	M	27	no	3	3	M.Sc.
E	F	27	no	5	5	M.Sc.
F	M	49	yes	23	23	M.Sc.
G	M	35	yes	10	11	M.Sc.
H	M	29	yes	3	3	M.Sc.
I	M	39	yes	11	15	M.Sc.
J	M	26	yes	4	4	M.Sc.

**Table 2:** Measurement results of initial tensile adhesion strength of CTA [N/mm^2^], model of failure, and calculated values of the series mean and standard deviation values.

Measurement/Operator	A	B	C	D	E	F	G	H	I	J
1	1.8	1.2	1.3	1.3	1.6	1.5	1.0	1.4	1.5	1.5 *
	AF-T	AF-T	AF-T	AF-T	AF-T	AF-T	AF-T	AF-T	AF-T	CF-A
2	1.7	1.2	1.2	1.6	1.5	1.3	1.3	1.6	1.4	1.0
	AF-T	AF-T	AF-T	AF-T	AF-T	AF-T	AF-T	AF-T	AF-T	AF-T
3	1.8	1.0	1.5	1.3	1.3	1.3	1.6 *	1.6	1.6	1.3
	AF-T	AF-T	AF-T	AF-T	AF-T	AF-T	AF-T	AF-T	AF-T	AF-T
4	1.7	1.4	1.4	1.5	1.7	1.6	1.2	1.5	1.4	1.4
	AF-T	AF-T	AF-T	AF-T	AF-T	AF-T	AF-T	AF-T	AF-T	AF-T
5	1.6	1.5	1.2	1.5	1.4	1.3	1.8 *	1.3	1.6	1.3
	AF-T	AF-T	AF-T	AF-T	AF-T	AF-T	AF-T	AF-T	AF-T	AF-T
6	1.6	1.4	1.6	1.4	1.3	1.3	1.1	1.5	1.3	1.1
	AF-T	AF-T	AF-T	AF-T	AF-T	AF-T	AF-T	AF-T	AF-T	AF-T
7	1.5	1.3	1.3	1.1 *	1.5	1.5	0.9 *	1.5	1.2	1.1
	AF-T	AF-T	AF-T	AF-T	AF-T	AF-T	AF-T	AF-T	AF-T	AF-T
8	1.6	1.1	1.3	1.5	1.5	1.4	1.2	1.4	1.6	1.2
	AF-T	AF-T	AF-T	AF-T	AF-T	AF-T	AF-T	AF-T	AF-T	AF-T
9	1.3	1.2	1.3	1.7	1.5	1.5	1.2	1.3	1.6	1.2
	AF-T	AF-T	AF-T	AF-T	AF-T	AF-T	AF-T	AF-T	AF-T	AF-T
10	1.2 *	1.2	1.5	1.5	1.4	1.4	1.0	1.4	1.3	1.2
	AF-T	AF-T	AF-T	AF-T	AF-T	AF-T	AF-T	AF-T	AF-T	AF-T
*m* (mean)	1.62	1.25	1.36	1.44	1.47	1.41	1.14	1.45	1.45	1.23
*s_D_* [N/mm^2^]	0.16	0.15	0.13	0.17	0.13	0.11	0.11	0.11	0.15	0.15
*s_D_*/*m* [%]	9.9	12.0	9.6	8.8	8.8	7.8	9.6	7.6	10.3	10.0
*s_D_*/*m* [%]	9.8					9.1				
*s_D_*/*m* [%]	9.4									

*—rejected value as required by clause 9 of EN 1348:2007; AF-T—adhesive failure between tile and adhesive; CF-A—cohesive failure within the adhesive.

**Table 3:** Measurement results of tensile adhesion strength after immersion in water of CTA [N/mm^2^], model of failure, and calculated values of the series mean and standard deviation values.

Measurement/Operator	A	B	C	D	E	F	G	H	I	J
1	1.2 *	0.7	0.9	0.9	0.9	1.0	0.8	1.1	0.9	0.9
	AF-T	AF-T	AF-T	AF-T	AF-T	AF-T	AF-T	AF-T	AF-T	AF-T
2	0.7 *	0.7	0.8	0.8	0.7	0.9	1.3 *	0.9	0.9	1.0
	AF-T	AF-T	AF-T	AF-T	AF-T	AF-T	AF-T	AF-T	AF-T	AF-T
3	0.7 *	0.6	0.8	0.8	0.8	0.9	0.7 *	0.9	0.8	0.9
	AF-T	AF-T	AF-T	AF-T	AF-T	AF-T	AF-T	AF-T	AF-T	AF-T
4	1.1	0.6	0.9	0.9	0.6 *	0.9	1.2	0.8	1.0	0.9
	AF-T	BT	AF-T	AF-T	AF-T	AF-T	AF-T	AF-T	AF-T	AF-T
5	1.0	0.4 *	1.0	0.9	0.8	0.8	1.1	1.0	1.0	1.0
	AF-T	BT	BT	AF-T	AF-T	BT	AF-T	AF-T	AF-T	AF-T
6	0.9	0.6	0.8	1.0	0.8	0.9	1.0	0.6 *	0.9	1.2 *
	AF-T	AF-T	BT	AF-T	AF-T	BT	AF-T	AF-T	AF-T	AF-T
7	1.0	0.5	1.0	1.0	1.0	1.0	1.0	0.8	1.0	1.0
	AF-T	AF-T	AF-T	AF-T	AF-T	AF-T	AF-T	AF-T	AF-T	AF-T
8	0.8	0.6	1.0	1.0	1.0	0.9	1.0	0.8	0.8	1.0
	AF-T	AF-T	AF-T	AF-T	AF-T	AF-T	AF-T	AF-T	AF-T	AF-T
9	0.9	0.5	0.8	1.0	1.0	0.8	1.0	0.8	0.9	0.8
	AF-T	AF-T	AF-T	AF-T	AF-T	AF-T	AF-T	AF-T	AF-T	AF-T
10	1.0	0.6	0.9	1.0	0.9	0.9	1.0	0.7	1.0	0.4 *
	AF-T	AF-T	AF-T	AF-T	AF-T	AF-T	AF-T	AF-T	AF-T	AF-T
*m* (mean)	0.96	0.60	0.89	0.93	0.88	0.90	1.01	0.87	0.92	0.94
*s_D_* [N/mm^2^]	0.10	0.07	0.09	0.08	0.11	0.07	0.11	0.12	0.08	0.07
*s_D_*/*m* [%]	10.4	11.7	10.1	8.6	12.5	7.8	10.9	13.8	8.7	7.4
*s_D_*/*m* [%]	10.7					9.7				
*s_D_*/*m* [%]	10.2									

*—rejected value as required by clause 9 of EN 1348:2007; AF-T—adhesive failure between tile and adhesive; BT—adhesive failure between tile and pull head plate.

**Table 4:** Measurement results of tensile adhesion strength after heat aging of CTA [N/mm^2^], model of failure, and calculated values of the series mean and standard deviation values.

Measurement/Operator	A	B	C	D	E	F	G	H	I	J
1	1.2	1.1	0.9	1.0	1.1	0.9	1.2	1.1	1.2	1.2
	AF-T	AF-T	AF-T	AF-T	AF-T	AF-T	AF-T	AF-T	AF-T	CF-A
2	0.8 *	1.2	0.9	1.0	1.1	1.0	1.5	1.4	1.3	1.0
	AF-T	AF-T	AF-T	AF-T	AF-T	AF-T	AF-T	AF-T	AF-T	CF-A
3	1.3 *	1.1	0.8	1.1	0.8	1.1	1.1	1.4	1.1	1.2
	AF-T	AF-T	AF-T	AF-T	AF-T	AF-T	AF-T	AF-T	AF-T	CF-A
4	1.0	1.2	0.8	1.0	1.0	1.0	1.2	1.2	1.4	1.1
	AF-T	AF-T	AF-T	AF-T	AF-T	AF-T	AF-T	AF-T	AF-T	AF-T
5	0.8 *	1.2	1.0 *	1.1	1.1	1.2	1.2	1.1	1.4	1.1
	AF-T	AF-T	AF-T	AF-T	AF-T	AF-T	AF-T	AF-T	CF-AAF-T	AF-T
6	0.9	1.2	0.6 *	1.3	1.0	1.0	1.2	1.2	1.0	1.2
	AF-T	AF-T	AF-T	AF-T	AF-T	AF-T	AF-T	AF-T	AF-T	CF-A
7	1.3 *	0.5 *	0.7	1.1	1.1	1.2	1.6 *	1.3	1.0	1.1
	AF-T	AF-T	AF-T	AF-T	AF-T	AF-T	AF-T	AF-T	AF-T	CF-A
8	1.0	1.3	0.8	1.0	0.5 *	1.3	1.3	1.1	1.3	1.0
	AF-T	AF-T	AF-T	AF-T	AF-T	AF-T	AF-T	AF-T	AF-T	CF-A
9	1.3 *	1.0	0.7	1.3	1.0	1.2	1.5	1.2	1.1	1.1
	AF-T	AF-T	AF-T	AF-T	AF-T	AF-T	AF-T	AF-T	AF-T	CF-A
10	1.1	1.3	0.8	1.4 *	0.8	1.1	1.4	1.3	1.2	1.1
	AF-T	AF-T	AF-T	AF-T	AF-T	AF-T	AF-T	AF-T	AF-T	CF-A
*m* (mean)	1.04	1.18	0.80	1.10	1.00	1.10	1.29	1.23	1.20	1.11
*s_D_* [N/mm^2^]	0.11	0.10	0.08	0.12	0.12	0.12	0.15	0.12	0.15	0.07
*s_D_*/*m* [%]	10.6	8.5	10.0	10.9	12.0	10.9	11.6	9.8	12.5	6.3
*s_D_*/*m* [%]	10.4					10.2				
*s_D_*/*m* [%]	10.3									

*—rejected value as required by clause 9 of EN 1348:2007; AF-T—adhesive failure between tile and adhesive; CF-A—cohesive failure within the adhesive.

**Table 5:** Measurement results of tensile adhesion strength after freeze–thaw cycles of CTA [N/mm^2^], model of failure, and calculated values of the series mean and standard deviation values.

Measurement/Operator	A	B	C	D	E	F	G	H	I	J
1	1.8	1.7	1.7	2.4 *	1.8	1.3	1.6	1.6	1.4	1.7
	CF-A	CF-A	CF-A	CF-A	CF-A	CF-A	CF-A	CF-A	CF-A	CF-A
2	1.8	1.7	1.8	2.2 *	1.8	1.6	2.2	1.7	1.6	1.6
	CF-A	CF-A	CF-A	CF-A	CF-A	CF-A	CF-A	CF-A	CF-A AF-T	CF-A
3	1.9	1.9	2.0	2.3 *	1.5	1.6	1.9	1.7	1.4	1.7
	CF-A	CF-A	CF-A	CF-A	CF-A	CF-A	CF-A	CF-A	CF-A AF-T	CF-A
4	2.0	2.1	1.7	1.2 *	1.9	1.7	2.0	1.8	1.4	1.6
	CF-A	CF-A	CF-A	BT	CF-A	CF-A	CF-A	CF-A	CF-A	CF-A
5	2.1	1.9	1.9	2.1	1.7	1.8	1.7	1.5	1.5	1.9
	CF-A	CF-A	CF-A	CF-A	CF-A	CF-A	CF-A	CF-A	CF-A	CF-A
6	2.1	1.6	1.7	0.7 *	2.0	1.6	1.9	2.0	1.7	1.7
	CF-A	CF-A	CF-A	BT	CF-A	CF-A	CF-A	CF-A	CF-A	CF-A
7	1.9	1.6	1.7	1.6	2.1	1.6	1.7	1.7	1.5	1.5
	CF-A	CF-A	CF-A	CF-A	CF-A	CF-A	CF-A	CF-A	CF-A	CF-A
8	1.2 *	1.4 *	1.9	1.9	1.9	1.6	1.8	2.0	1.7	1.7
	AF-T	CF-A	CF-A	CF-A	CF-A	CF-A	CF-A	CF-A	CF-A	CF-A
9	2.2	1.9	2.0	1.2 *	1.8	1.6	2.2	1.5	1.9 *	1.5
	CF-A	CF-A	CF-A	BT	CF-A	CF-A	CF-A	CF-A	CF-A	CF-A
10	2.0	2.0	1.7	2.2 *	1.8	1.5	1.9	1.8	1.4	1.7
	CF-A	CF-A	CF-A	CF-A	CF-A	CF-A	CF-A	CF-A	CF-A AF-T	CF-A
*m* (mean)	1.98	1.82	1.81	NC	1.83	1.59	1.89	1.73	1.55	1.66
*s_D_* [N/mm^2^]	0.14	0.18	0.13		0.16	0.13	0.20	0.18	0.17	0.12
*s_D_*/*m* [%]	7.1	9.9	7.2		8.7	8.2	10.6	10.4	8.6	7.2
*s_D_*/*m* [%]	8.2					9.0				
*s_D_*/*m* [%]	8.7									

*—rejected value as required by clause 9 of EN 1348:2007; NC—not calculated; AF-T—adhesive failure between tile and adhesive; CF-A—cohesive failure within the adhesive; BT—adhesive failure between tile and pull head plate.

**Table 6:** General mean, repeatability, and reproducibility of initial tensile adhesion strength, tensile adhesion strength after immersion in water, tensile adhesion strength after heat aging, and tensile adhesion strength after freeze–thaw cycles calculated according to ISO 5725:1994+AC1:2002.

Measurement	$\hat{m}$	*s_r_*	*s_R_*
initial adhesion strength	1.39	0.14	0.19
tensile adhesion strength after immersion in water	0.89	0.09	0.14
tensile adhesion strength after heat aging	1.11	0.12	0.18
tensile adhesion strength after freeze–thaw cycles	1.76	0.15	0.21

## Data Availability

Not applicable.
